# Impact of Adverse Childhood Experiences on Older Adult Poverty: Mediating Role of Depression

**DOI:** 10.3389/fpubh.2021.749640

**Published:** 2021-11-05

**Authors:** Hui Liao, Chaoyang Yan, Ying Ma, Jing Wang

**Affiliations:** ^1^Department of Health Management, School of Medicine and Health Management, Tongji Medical College, Huazhong University of Science and Technology, Wuhan, China; ^2^The Key Research Institute of Humanities and Social Science of Hubei Province, Huazhong University of Science and Technology, Wuhan, China; ^3^Institute for Poverty Reduction and Development, Huazhong University of Science and Technology, Wuhan, China

**Keywords:** adverse childhood experiences, family dysfunction, older adult poverty, depression, mediating effect

## Abstract

**Background:** Adverse childhood experiences (ACEs) refer to traumatic events experienced by children in early life, including abuse, neglect, and family dysfunction, which are common worldwide. ACEs are harmful to mental health, and psychological problems can influence personal economic poverty in adulthood. We focused on family dysfunction and discussed the effect of different types of ACEs on poverty and the corresponding mediating effect of depression.

**Materials and Methods:** A total of 9,910 individuals who were 60 years or older from the China Health and Retirement Longitudinal Study in 2014 and 2015 were analysed. The chi-square test was used to compare poverty incidence among subgroups of independent or control variables. Binary logistic regression analysis was used to test the effect of different types of ACEs on depression, and four logistic regression models were established to observe the association between ACEs on older adult poverty and the mediating effect of depression. The path diagram of the direct effect and indirect effect was drawn to test the mediating effect of depression.

**Results:** Early death of father, the male guardian getting upset and witnessing violence of father to mother are the risk factors for older adult poverty, whereas female guardian getting upset, relationship with female guardians and parental quarrel are protective factors for older adult poverty. Furthermore, depression has a partial mediating effect on some factors including early death of father, male guardian getting upset, relationship with female guardian, parental quarrel, and witnessing violence of father to mother.

**Conclusions:** Paternal ACE factors can directly make children more likely to fall into poverty as older adults and can indirectly influence older adult poverty through the partial mediating effect of depression. Assisting poor families, providing psychological counselling, formulating family visit plans, nurturing orphan children under state supervision, and other policies that focus on groups that have experienced paternal ACE events are essential to eliminating the risk factors that influence older adult poverty.

## Introduction

Adverse childhood experiences (ACEs) refer to traumatic and frequent events that children suffer early in life. ACEs defined by Dube et al. include five forms of child abuse (physical abuse, sexual abuse, emotional abuse, emotional negligence, and physical negligence) and five family challenges (parental divorce, violence toward mother, family mental health problems, family substance use, and family imprisonment) (1). According to Kim et al. and Felitti et al., ACEs are mainly divided into three aspects: abuse, neglect, and family dysfunction. Abuse includes physical, emotional, and sexual abuse; neglect includes physical and emotional neglect; and family dysfunction includes early death of parent, parental divorce, witnessing inter-parental violence (IPV), crimes by family members, alcohol, and drug abuse (2, 3). Abuse and neglect can come from the subjects of personal contact in the environment of home, school and society, whereas family dysfunction focuses on the negative effect of family environment and family members on personal health.

ACEs are common worldwide. In the United States, 60.9% of adults have experienced at least one form of ACEs, and 15.6% of adults have experienced four or more forms of ACEs (4). A prevalence study in England showed that approximately half of adults report experiencing at least one form of ACEs (5). In Ho et al.'s research about Chinese young people, nearly 75% of the participants reported at least one ACE, and 31% reported three or more ACEs (6). The exposure of ACEs is widespread in China, with roughly three in four Chinese adolescents experiencing at least one form of ACE during childhood (7).

Different types of ACEs cause different health damages. Abuse mainly focuses on physical violence and brings direct physical trauma. Neglect focuses on emotional harm and is more likely to lead to psychological imbalance and the formation of risky behaviours. For example, a survey confirmed that neglect increased the incidence of drug and alcohol abuse among teenagers (8). By contrast, family dysfunction usually has intergenerational influence and a wider scope of destruction (9). Given that family dysfunction has a high incidence and a variety of forms, studying whether its diverse presentation forms have any difference in health damage is especially worthwhile.

Among health damage related with ACEs, mental illness is common. The association between exposure to ACEs and the development of depression during adulthood has been addressed by previous studies. Blum et al. suggested that ACEs exposure is a strong antecedent related to depressive symptoms (10). Anda et al. showed the risk of depression in adulthood increases as the number of reported adverse experiences increases (11). Al Shawi found most forms of ACEs especially abuse, neglect are associated with depression (12), and studies have demonstrated that children who experienced different kinds of violence are more likely to suffer emotional and neurological deficits that may occur not only during childhood but also later in life (13, 14). Weich et al. confirmed that the experience of family dysfunction in childhood is related to the increased risk of later developing mental illness, with the greatest risk of depression (15).

Depression also would worsen economic consequences for individuals and impose huge economic costs on the society. It directly affecting the way people think by capturing their attention and distorting their memory (16). Such effects are likely to influence economic preferences and beliefs, thereby distorting important economic decisions made by individuals, such as how much to work, invest and consume (17). Reduced concentration and greater fatigue reduce work productivity and lower incomes. In addition, mental illness may hinder education and skill acquisition among youth; hence, personal social skills and work quality are weakened, thus increasing the likelihood of poverty (18). As for the elderly people, depression is one of the most serious problems, which erodes their health and quality of life. It is reported that the additional medical cost per one depressed older adult is USD 686 for 1 year and USD 5271 for 4 years (19), thereby having adverse impact on economic condition in the long term.

Evidence has shown that ACEs have a potential association with poverty. For example, Metzler et al. found that compared with participants with no ACE, those with higher ACE scores were more likely to report high school non-completion, unemployment and living in a household below the poverty level (20). Adults reporting histories of child abuse and neglect have been shown to have lower levels of education, lower employment earnings, and fewer assets than matched controls (21). Multiple types of child abuse have been shown to negatively affect adult employment status (22) and have been linked to poverty (23). Thus, studying whether ACEs affect poverty directly or influence poverty through mental health, deserves attention.

Considering possible differences in the association between different types of ACEs and older adult poverty, this study focuses on family dysfunction. The purpose of this study is two-fold: (1) to examine how different types of ACEs contribute to the risk of older adult poverty and (2) to analyse the mediating effect of depression on ACEs influencing older adult poverty in a sample of elderly people aged 60 and above in China.

Previous studies on ACEs mostly have explored the incidence of ACEs in different populations, regions, or races. The studies focused on the impact of ACEs on public health and the economy. However, previous studies mostly focused on abuse and neglect; only a few studies tackled family dysfunction. Besides, ACEs were rarely explored in connexion with health and economic factors at the same time. Therefore, this study will pay attention to family dysfunction and include ACEs, mental health and economy to explore the association between ACEs and older adult poverty, as well as whether depression has a mediating effect in the process of ACEs affecting older adult poverty. Supporting early intervention is helpful in the prevention of poverty from the family and psychological aspects.

## Materials and Methods

### Data Source and Sample Selection

The data used in this research were obtained from the China Health and Retirement Longitudinal Study (CHARLS) in 2014 and 2015. CHARLS collects the health and ageing status of the middle-aged and elderly population aged 45 and over in China. The national baseline survey of CHARLS was conducted in 2011, covering 150 counties and 450 villages. A follow-up survey is conducted every 2–3 years. At present, CHARLS has fulfilled regular surveys in 2011, 2013, 2015, and 2018.

The regular survey adopts multi-stage sampling, and the probability proportional to size (PPS) sampling method is adopted in the sampling stage of counties/districts and villages. A total of 150 districts and counties were randomly selected from 30 provincial administrative units across the country (excluding Tibet, Taiwan, Hong Kong, and Macao), then three villages or communities were randomly selected from each of the above 150 districts and counties. A total of 450 villages/communities were ultimately obtained. By the time of the national follow-up in 2015, the sample size included 23,000 individuals from 12,400 households. The questionnaire of the CHARLS regular survey contains information regarding basic demographics, family structure and financial support, health status, health care, employment, and household economy (income, consumption, and wealth) and the basic situation of the community.

CHARLS also conducted the “Chinese Residents” Life Course Survey' in 2014, whereby the sampling method is the same as that of regular surveys, and it also uses multi-stage sampling and PPS sampling. Its questionnaire content is different. In addition to the questionnaire content of regular surveys, it includes childhood social and economic status, childhood neighbourhood quality, childhood friendship and experience, criminal activity, parent mental health, and relationship with parents. The 2014 survey sample was 20,547 individuals, and a total of 20,284 individual panel data were covered by the 2014 and 2015 surveys.

In this study, 9,910 individuals aged 60 years old and over covered by the 2014 and 2015 surveys were analysed after excluding 364 cases with missing key variables regarding family dysfunction.

### Variables

#### Dependent Variable

The main dependent variable is older adult poverty, and the data about dependent variable are obtained from the Harmonised CHARLS, which is a user-friendly version of a subset of the CHARLS interviews. Harmonised CHARLS was created by the USC Gateway to Global Ageing Data team to make the data more accessible to researchers and to facilitate comparisons among different waves. Older adult poverty is measured by the annual household income per capita according to the total household income and number of family population in 2015. China's national poverty line is the 2011 per capita net income of 2,300 CNY. After the Consumer Price Index adjustment, the national poverty line is converted to 2,391 CNY in 2015. According to the US$ average exchange rate in 2015 (1 USD = 6.23 CNY), 2,391 CNY is equivalent 384 USD. In this study, annual household income per capita below 384 USD is defined as older adult poverty. The total household income and number of family population are directly obtained from the Harmonised CHARLS database, where the total household income is the sum of all income at the household level, including earning income, capital income, pension income, income from government transfers, other sources of income, and the total income from other household members. The family population includes the respondent, spouse, parents (including biological parents, stepparents, and adoptive parents), parents-in-law, children, siblings, siblings-in-law, and other household members.

#### Independent Variables

On the basis of a large body of literature on family dysfunction events, this study selected 10 independent variables reflecting different types of family dysfunction from the family information module of CHARLS 2014. They were early death of mother, early death of father, parental divorce, female guardian getting upset, male guardian getting upset, relationship with female guardian, relationship with male guardian, parental quarrel, and witnessing IPV.

#### Mediating Variable

In this study, depression was assumed as the mediating variable, and the data come from the depression scale of CHARLS 2015 data. CHARLS includes the Depression Scale of the Centre for Epidemiology to design 10 items. The items are (1) I was bothered by things that do not usually bother me. (2) I had trouble keeping my mind on what I was doing. (3) I felt depressed. (4) I felt everything I did was an effort. (5) I felt hopeful about the future. (6) I felt fearful. (7) My sleep was restless. (8) I was happy. (9) I felt lonely. (10) I could not get “going.” Respondents reported the frequency of occurrence of each item during the past week, and the frequency of each item was set to 0–3. The total score corresponding to the 10 items is 0–30. According to Andresen et al. (24), a total score of depressive symptoms ≤10 is considered normal, whereas a score of 10 or higher is considered a depressive state. The higher the score, the more severe the depression. Thus, depression in this study is a classified variable.

#### Control Variables

Control variables in this study were from the demographic background part of CHARLS 2015, which includes gender, age, education, marital status, and residence. Table 1 shows the detailed definition and coding of each control variable.

**Table 1:** Code and question description of variables.

**Variables**	**Code**	**Question description**
Gender	0 = male, 1 = female	Interviewer recorded R's gender
Age	0 = 60–75, 1 ≥ 75	What is your actual date of birth
Education	0 = no formal education, 1 = elementary school and below, 2 = middle school, 3 = high school and above	Has your highest level of education changed from the last wave? If so, what is the highest level of education you have attained now (not including adult education)?
Marital status	0 = married, 1 = unmarried	What is your marital status?
Residence	0 = urban community, 1 = rural village	Was the type of address village or city/town?
Early death of parents	0 = parent died after respondent was age 16 or still alive, 1 = parent died before respondent was age 16	Is your biological mother/father alive? In what year did she/he pass away?
Parental divorce	0 = parent divorced after respondent was age 16 or still a couple, 1 = parent divorced before respondent was age 16	Were your biological parents divorced? What was your age when your parents divorced?
Female/male guardian getting upset	0 = no (a little of the time), 1 = yes (some of the time, good part of the time, most of the time)	During your childhood, did your female/male guardian get upset easily or feel panicky?
Relationship with female/male guardian	0 = poor, 1 = good (fair, good, very good, excellent)	How would you rate your relationship with your female/male guardian when you were growing up?
Parental quarrel	0 = no (never), 1 = yes (not very often, sometimes, often)	Did your parents often quarrel?
Witnessing inter-parental violence (IPV)	0 = no (never), 1 = yes (not very often, sometimes, often)	Has your mother/father ever beat up your father/mother?
Depression	0 = no, 1 = yes	
Older adult poverty	0 = no, 1 = yes	

### Statistical Analysis

Firstly, poverty incidence was compared among subgroups of independent or control variables by conducting the chi-square test. Secondly, binary logistic regression analysis was used to show the effect of different types of ACEs on depression. Then, the association between ACEs on older adult poverty and the mediating effect of depression were analysed by using four logistic regression models. Finally, a path analysis was constructed to yield the effect values and calculate the direct, indirect, and total effects of ACEs on older adult poverty. On these bases and according to the rules of mediating effect judgement, the mediating effect of depression in the process of ACEs affecting older adult poverty was tested.

According to Baron and Kenny (25), a mediation model needs to fulfil the following criteria. (1) The independent variable (e.g., ACEs) should be significantly associated with the dependent variable (e.g., older adult poverty). (2) The independent variable should be significantly associated with the mediating variable (e.g., depression). (3) The mediating variable should be significantly associated with the dependent variable when the independent variable is included in the same model. (4) The independent variable must be known to cause the mediating variable, which, in turn, causes the dependent variable.

Furthermore, the mediating effect of depression on ACEs and older adult poverty should be judged on the basis of the following rules, according to Zhonglin (26). (1) If the indirect effect of an independent variable is significant, but its direct effect is not significant, then depression has a complete mediating effect on the factor. (2) If the indirect and direct effects of an independent variable are both significant, and the indirect effect and total effect are both either positive or negative, then depression has a partial mediating effect. (3) If the indirect and direct effects of an independent variable are both significant, but the indirect effect and total effect are opposite, then depression is not a mediator for the factor.

Most analyses were performed using SPSS12, and the path diagram is drawn using AMOS 23. The significant level for Type I Error was set at α = 0.05 with *p* < 0.05 considered statistically significant.

### Ethics Statement

The CHARLS study data are publicly available and open to researchers worldwide. Ethics approval for data collection in CHARLS was obtained from the Biomedical Ethics Review Committee of Peking University, and the Ethics Committee of Tongji Medical College, Huazhong University of Science and Technology approved the research proposal. All respondents were required to sign consent under conditions of privacy after clarifying that the decision to participate in the research was entirely voluntary.

## Results

Table 2 shows older adult poverty incidence difference within subgroups of independent or control variables. In total, the number of male and female participants was almost the same, with male respondents accounting for 49.2% and female respondents accounting for 50.8%, and most of them were 60–75 years old (82.7%). Only 7.4% of the participants attained high school or above, and more participants lived in rural areas (60.3%) and were married (78.4%). Among ACEs, the incidence of early death of father (13.1%) is slightly higher than that of mother (8.6%). The incidence of female guardian getting upset (45.8%) is much higher than that of male guardian (17.2%). In a comparison of incidences of witnessing violence of mother to father (3.6%), that of father to mother is much higher (14.9%). In addition, most of the participants answered that they had a good relationship with their guardians when they were children (99.0%, 98.8%). The incidence of parental divorce is extremely low (0.9%), whereas the rate of quarrel is relatively high (61.8%).

**Table 2:** Older adult poverty incidence within subgroups of independent or control variables (*n* = 9,910).

**Item**		**Total**	**Older adult poverty** ***n*** **(%)**		**χ^**2**^**	***p***
		***n* (%)**	**Yes**	**No**		
Control variables	Gender				0.1	0.743
	Male	4,880 (49.2)	1,080 (22.1)	3,800 (77.9)		
	Female	5,030 (50.8)	1,127 (22.4)	3,903 (77.6)		
	Age				25.0	<0.001
	60-75	8,193 (82.7)	1,903 (23.2)	6,290 (76.8)		
	>75	1,717 (17.3)	304 (17.7)	1,413 (82.3)		
	Education				93.6	<0.001
	no formal education	3,476 (35.1)	865 (24.9)	2,611 (75.1)		
	elementary school and below	4,381 (44.2)	1,019 (23.3)	3,362 (76.7)		
	middle school	1,321 (13.3)	250 (18.9)	1,071 (81.1)		
	high school and above	732 (7.4)	73 (10.0)	659 (90.0)		
	Marital status				29.1	<0.001
	Married	7,772 (78.4)	1,639 (21.1)	6,133 (78.9)		
	Unmarried	2,138 (21.6)	568 (26.6)	1,570 (73.4)		
	Residence				369.0	<0.001
	urban community	3,930 (39.7)	486 (12.4)	3,444 (87.6)		
	rural village	5,980 (60.3)	1,721 (28.8)	4,259 (71.2)		
Independent variables	Early death of mother				9.4	<0.01
	No	9,055 (91.4)	1,981 (21.9)	7,074 (78.1)		
	Yes	855 (8.6)	226 (26.4)	629 (73.6)		
	Early death of father				22.4	<0.001
	No	8,609 (86.9)	1,851 (21.5)	6,758 (78.5)		
	Yes	1,301 (13.1)	356 (27.4)	945 (72.6)		
	Parental divorce				1.8	0.184
	No	9,825 (99.1)	2,183 (22.2)	7,642 (77.8)		
	Yes	85 (0.9)	24 (28.2)	61 (71.8)		
	Female guardian getting upset				45.9	<0.001
	No	5,371 (54.2)	1,336 (24.9)	4,035 (75.1)		
	Yes	4,539 (45.8)	871 (19.2)	3,668 (80.8)		
	Male guardian getting upset				30.6	<0.001
	No	8,210 (82.8)	1,742 (21.2)	6,468 (78.8)		
	Yes	1,700 (17.2)	465 (27.4)	1,235 (72.6)		
	Relationship with female guardian				11.8	<0.01
	Poor	95 (1.0)	35 (36.8)	60 (63.2)		
	Good	9,815 (99.0)	2,172 (22.1)	7,643 (77.9)		
	Relationship with male guardian				2.8	0.096
	Poor	115 (1.2)	33 (28.7)	82 (71.3)		
	Good	9,795 (98.8)	2,174 (22.2)	7,621 (77.8)		
	Parental quarrel				47.0	<0.001
	No	3,781 (38.2)	980 (25.9)	2,801 (74.1)		
	Yes	6,129 (61.8)	1,227 (20.0)	4,902 (80.0)		
	Witnessing violence of father to mother				26.7	<0.001
	No	8,438 (85.1)	1,803 (21.4)	6,635 (78.6)		
	Yes	1,472 (14.9)	404 (27.4)	1,068 (72.6)		
	Witnessing violence of mother to father				9.5	<0.01
	No	9,554 (96.4)	2,104 (22.0)	7,450 (78.0)		
	Yes	356 (3.6)	103 (28.9)	253 (71.1)		

Older adult poverty is significantly related to age, education, marital status, and residence (*p* < 0.001). The poverty incidence is higher for people aged 60–75, indicated by lower education, single status and residence in a rural village; whereas gender and older adult poverty are not statistically significant (*p* = 0.743). Among the family dysfunction variables, older adult poverty prevalence of those with early death of parents, male, and female guardians getting upset, relationship with female guardian, parental quarrel, and witnessing IPV in childhood is statistically significant. Specifically, the poverty incidence is higher for those who have experienced early death of father (27.4%) or mother (26.4%). When a male guardian gets upset, his children have a higher poverty incidence in adulthood (27.4%). Children with a poor relationship with their female guardian have a higher poverty incidence when they become older adults (36.8%). Children who witnessed the violence of father to mother and mother to father have a higher older adult poverty incidence, at 27.4 and 28.9%, respectively.

Table 3 tests the association between ACEs and depression. It shows that early death of father, guardians being upset, relationship with guardians, parental quarrel, and witnessing violence of father to mother were significantly related to the depression of children who experienced these ACE events (*p* < 0.05). Among the significant independent variables, early death of father, guardians being upset and witnessing the violence of father to mother are risk factors because their OR > 1; whereas the relationship with guardians and parental quarrel are protective factors for their OR <1. Thus, those who experienced early death of father are 1.3 times more likely to be depressed than those who did not experience early death of father. Those whose female and male guardians got upset are, respectively, 1.1 and 1.8 times more likely to be depressed than those whose female and male guardians did not. Those who witnessed violence of their father to mother are 1.4 times more likely to be depressed than those who did not. Such results mean that children who have experienced early death of father, whose guardians got upset, whose relationship with their guardian was poor, whose parents did not quarrel and who witnessed the violence of father to mother are more likely to be depressed.

**Table 3:** Associations between ACEs and depression.

**Variables**		**OR**	**95% CI**
Early death of mother (Ref no.)	Yes	1.1	0.96–1.31
Early death of father (Ref no.)	Yes	1.3^***^	1.12–1.45
Parental divorce (Ref no.)	Yes	1.2	0.78–1.98
Female guardian getting upset (Ref no.)	Yes	1.1^*^	1.01–1.24
Male guardian getting upset (Ref no.)	Yes	1.8^***^	1.60–2.05
Relationship with female guardian (Ref poor.)	Good	0.5^***^	0.30–0.71
Relationship with male guardian (Ref poor.)	Good	0.6^**^	0.37–0.82
Parental quarrel (Ref no.)	Yes	0.9^*^	0.81–0.99
Witnessing violence of father to mother (Ref no.)	Yes	1.4^***^	1.20–1.57–
Witnessing violence of mother to father (Ref no.)	Yes	1.1	0.90–1.47
Constant		1.2	

* *p < 0.05,*

** *p < 0.01,*

*** *p < 0.001*.

*OR (odds ratio) stands for the relative risk, the independent variable is a risk factor if OR > 1 or a protective factor if OR <1*.

*95% CI (95% Confidence Interval) denotes the confidence interval estimates the range of the OR-value with confidence level of 95%*.

Table 4 shows the influence of ACE factors on older adult poverty and the corresponding mediating effect of depression. Model 1 shows the association between ACEs and older adult poverty, and completes step 1 for building the mediation model in above statistical analysis. Early death of mother, early death of father, guardians being upset, relationship with female guardian, parental quarrel, witnessing violence of father to mother were significant in this model (*p* < 0.05). After adding the control variables to conduct a robustness test resulted in Model 2, the association between early death of mother and older adult poverty was not significant (*p* > 0.05), but early death of father, guardians being upset, relationship with female guardian, parental quarrel and witnessing violence of father to mother on older adult poverty were still significant. Among these factors, early death of father, male guardian getting upset and witnessing violence of father to mother are risk factors for their OR > 1; whereas female guardian getting upset, relationship with female guardian and parental quarrel are protective factors because their OR <1. Thus, those who have experienced early death of father are 1.3 times more likely to fall into older adult poverty than those who have not experienced early death of father. Moreover, those whose male guardians got upset are 1.5 times more likely to fall into older adult poverty than those whose male guardian did not get upset. Those who witnessed violence of father to mother are 1.3 times more likely to fall into older adult poverty than those who did not. Model 3 added depression on the basis of Model 1. Model 3 shows that depression and older adult poverty are significantly associated (*p* < 0.001); hence, it completes step 3 for building the mediation model in the above statistical analysis. Adding the control variables to Model 3 for carrying out a robustness test resulted in Model 4. The association between depression and older adult poverty remained significant, and children with depression are 1.4 times more likely to become poor than those without depression. All of these results implied that children who have experienced early death of father, whose female guardians did not get upset, whose male guardians got upset, who had poor relationship with female guardians, whose parents did not quarrel and who witnessed violence of father to mother are more likely to be poor in adulthood. In addition, depression may be a mediator between these ACE factors and older adult poverty.

**Table 4:** Older adult poverty models on ACEs, depression, and control variables.

**Variables**		**Model 1**		**Model 2**		**Model 3**		**Model 4**	
		**OR**	**95% CI**	**OR**	**95% CI**	**OR**	**95% CI**	**OR**	**95% CI**
Early death of mother (Ref no.)	Yes	1.2^*^	1.02–1.42	1.2	0.97–1.36	1.2^*^	1.01–1.41	1.1	0.97–1.36
Early death of father (Ref no.)	Yes	1.4^***^	1.22–1.60	1.3^***^	1.15–1.52	1.43^***^	1.20–1.57	1.3^***^	1.13–1.50
Parental divorce (Ref no.)	Yes	1.5	0.91–2.40	1.4	0.87–2.34	1.5	0.90–2.37	1.4	0.86–2.33
Female guardian getting upset (Ref no.)	Yes	0.6^***^	0.57–0.72	0.6^***^	0.56–0.71	0.6^***^	0.56–0.71	0.6^***^	0.55–0.70
Male guardian getting upset (Ref no.)	Yes	1.7^***^	1.48–1.96	1.5^***^	1.32–1.77	1.6^***^	1.40–1.85	1.5^***^	1.27–1.70
Relationship with female guardian (Ref poor.)	Good	0.6^*^	0.36–0.88	0.6^*^	0.37–0.93	0.6^*^	0.39–0.96	0.6^*^	0.39–0.97
Relationship with male guardian (Ref poor.)	Good	0.9	0.59–1.42	0.9	0.57–1.41	1.0	0.63–1.51	0.9	0.60–1.47
Parental quarrel (Ref no.)	Yes	0.7^***^	0.67–0.83	0.8^***^	0.69–0.86	0.7^***^	0.67–0.83	0.8^***^	0.69–0.86
Witnessing violence of father to mother (Ref no.)	Yes	1.4^***^	1.20–1.62	1.3^***^	1.12–1.52	1.4^***^	1.17–1.57	1.3^**^	1.10–1.49
Witnessing violence of mother to father (Ref no.)	Yes	1.1	0.86–1.46	1.1	0.85–1.45	1.1	0.85–1.44	1.1	0.84–1.44
Gender (Ref male.)	Female			0.9	0.84–1.04			0.9^*^	0.80–0.99
Age (Ref 60–75.)	>75			0.6^***^	0.55–0.74			0.7^***^	0.57–0.76
Education (Ref no formal education.)	Elementary school and below			0.9	0.84–1.07			1.0	0.85–1.07
	Middle school			0.8	0.71–1.00			0.9	0.72–1.03
	High school and above			0.5^***^	0.39–0.66			0.5^***^	0.40–0.69
Marital status (Ref married.)	Unmarried			1.5^***^	1.34–1.71			1.5^***^	1.31–1.67
Residence (Ref urban community.)	Rural village			2.5^***^	2.26–2.84			2.5^***^	2.20–2.77
Depression (Ref no.)	Yes					1.6^***^	1.45–1.78	1.4^***^	1.27–1.57
Constant		0.6		0.4^**^		0.5^*^		0.3^***^	

* *p < 0.05,*

** *p < 0.01,*

*** *p < 0.001.*

*OR (odds ratio) stands for the relative risk, the independent variable is a risk factor if OR > 1 or a protective factor if OR <1*.

*95% CI (95% Confidence Interval) denotes the confidence interval estimates the range of the OR-value with confidence level of 95% Model 1 showed the association between ACEs and older adult poverty*.

*Model 2 added control variables based on Model 1*.

*Model 3 added depression based on Model 1*.

*Model 4 added control variables based on Model 3*.

**Figure 1:**
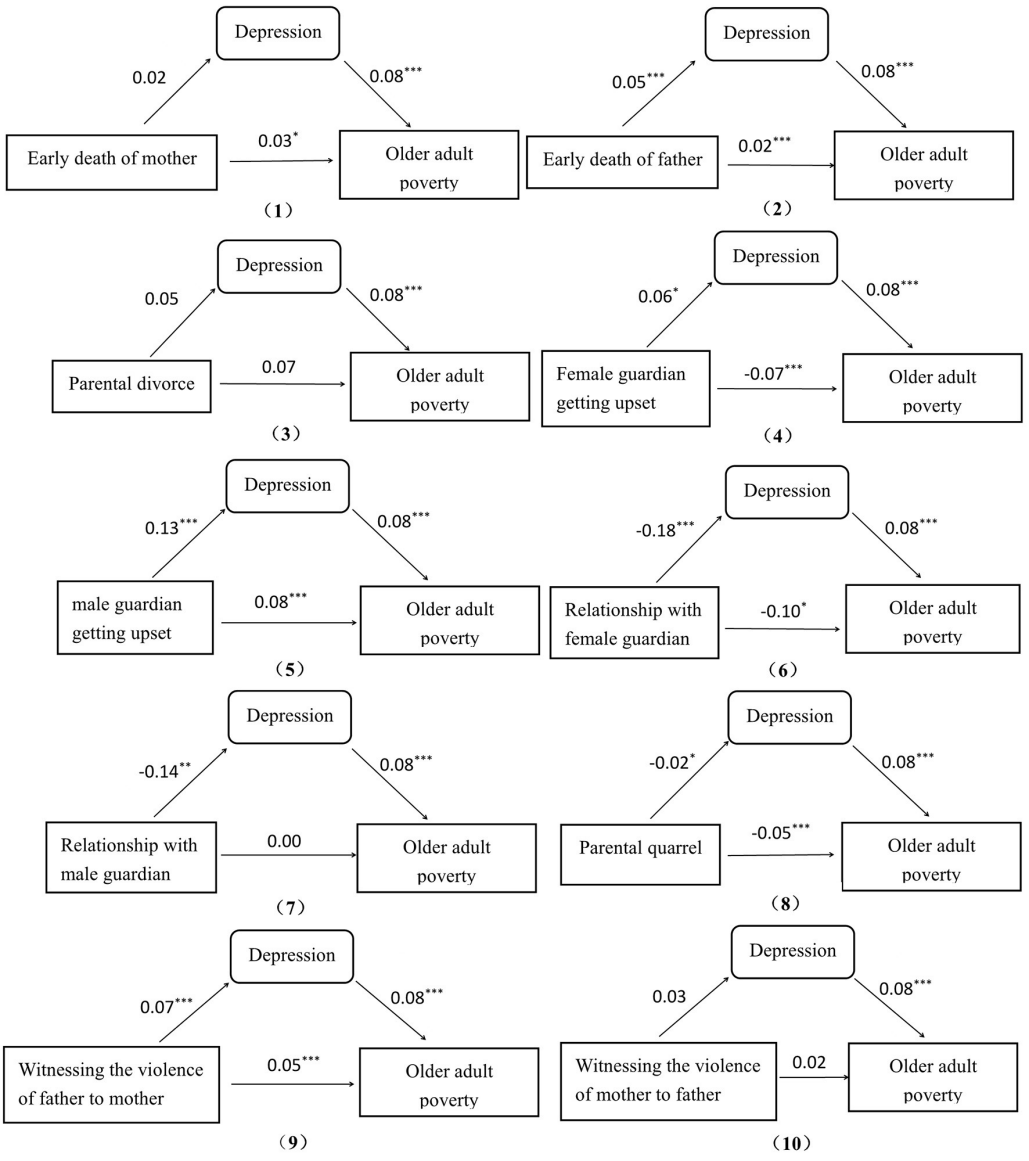
Path diagram of effects. *p < 0.05, **p < 0.01, ***p < 0.001. The effect values were output from a path analysis that took 10 ACE factors as exogenous variables, depression as a mediating variable and older adult poverty as the endogenous variable using AMOS 23. (2, 5, 6, 8, 9) indicate having a mediating effect and others do not.

Figure 1 shows the path diagram of the direct and indirect effects between different types of ACEs and older adult poverty by depression. The figure shows that 6 in 10 ACE factors have a direct effect on older adult poverty, namely, early death of father, female and male guardians getting upset, relationship with female guardian, parental quarrel, and witnessing violence of father to mother. These six ACE factors also have an indirect effect on older adult poverty through depression.

The figure also shows that depression has different mediating effects on these six ACE factors, but depression does not play a complete mediating role in any ACE factor. According to the criterion of mediating effect in method, depression has a partial mediating effect on five ACE factors, namely, early death of father, male guardians getting upset, relationship with female guardian, parental quarrel, and witnessing violence of father to mother. By contrast, depression is not a mediator for the remaining factors. Furthermore, among the five ACE factors in which depression plays a partial mediating role, early death of father, male guardians getting upset, and witnessing violence of father to mother are all only related to the father. Only one factor, namely, relationship with female guardian, is related to the mother.

Table 5 shows the proportions of depression mediating effect in total effect among the five ACE factors with significant mediation effects. According to Figure 1, the total effect is the sum of the direct effect (c) and the indirect effect, which is the product of the effect of the ACE factor on depression (a) and the effect of depression on older adult poverty (b). The proportion of mediating effect in total effect is the ratio of the indirect effect to the total effect. This finding shows that the total effect could be positive or negative. Although the absolute value of the total effect of relationship with female guardian to older adult poverty is maximum (0.1144) and the absolute value of the total effect of early death of father to older adult poverty is minimum (0.0240), the former is a protective factor because it is negative, whereas the latter is a risk factor for it is positive. Moreover, the proportion of the mediating effect on early death of father is the highest (16.7%), meaning that depression could be an important mediation between paternal ACEs and older adult poverty.

**Table 5:** Proportions of mediating effect in total effect.

**Path**	**Effect of the ACEs factor on depression (a)**	**Effect of depression on older adult poverty (b)**	**Direct effect (c)**	Indirect effect (*d* = a*b)	**Total effect (*t* = c + d)^#^**	**Proportion of mediating effect in total effect (%) (d/t)^#^**
Early death of father to older adult poverty	0.05	0.08	0.02	0.0040	0.0240	16.7
Male guardian getting upset to older adult poverty	0.13	0.08	0.08	0.0104	0.0904	11.5
Relationship with female guardian to older adult poverty	−0.18	0.08	−0.10	−0.0144	−0.1144	12.6
Parental quarrel to older adult poverty	−0.02	0.08	−0.05	−0.0016	−0.0516	3.1
Witnessing violence of father to mother to older adult poverty	0.07	0.08	0.05	0.0056	0.0556	10.1

# The total effect is a*b + c, and the proportion of mediating effect in total effect is (a*b)/(a*b + c).

## Discussion

Our results show that the ACEs affect people falling into poverty as older adults. People who have experienced early death of father, whose female guardians did not get upset, whose male guardians got upset, who had poor relationship with female guardians, whose parents did not quarrel and who witnessed their father hitting their mothers are more likely to fall into older adult poverty. Furthermore, depression plays a partial mediating role in some factors including early death of father, male guardian getting upset, relationship with female guardian, parental quarrel, and witnessing violence of father to mother, thereby influencing the associations between these factors and poverty to a certain extent. Those results imply the paternal ACE factors could directly influence older adult poverty and have a high probability of indirectly leading to older adult poverty through depression.

The possible reasons for the direct influence on older adult poverty could result from the father's economic status, which will influence the opportunities for children to compete and earned. The father is the main labour force in a family and contributes the main economic income to the family, so the absence of father due his death will greatly reduce the earning of the whole family. Firstly, financially struggling families cannot easily provide children with opportunities for better and longer-term education, and some children are forced to drop out of school. Thus, the children obtain less knowledge and less earning as they grow up. Rothon et al. and Baron et al. demonstrated a link between poor paternal support and lower educational attainment (27) as well as with economic adversity in adulthood (25). Secondly, some children whose family lacks money for their medical treatment are possibly physically weak or suffer from illnesses, so that such individuals would lack the ability to work and be poverty due to illness when they becoming older. This finding is similar to that of Sapkota et al.'s study, which found that patients in families on the edge of poverty often choose to avoid medical treatment, further impoverishing them (28).

The possible reasons of indirect influence on older adult poverty could result from psychological and emotional trauma; mental problems might evolve into depression, and depression makes people lose control of their emotions, increases medical expenses, or decreases normal interpersonal skills. Katz et al. found that men care less about their partners or children and use behavioural violence more frequently in the family (29). Fathers may be too busy to accompany their children or scold their children, because they want to establish the image of a majestic father or simply due to bad behaviour or poor mental states caused by alcohol, drug abuse, crime, and anxiety. Fathers often beat and scold their partners or children. Firstly, children who grow up in such environments are prone to psychological and emotional trauma. At the same time, depression worsens children's self-control and emotional adjustment abilities, and they lack correct value guidance from their fathers. Such children are more likely to commit rebellious behaviours, such as crimes, which affect their future development and even cause them to lose jobs. Secondly, severe depression requires medication and treatment costs. Long-term expenses will naturally increase financial burden due to depression being a chronic disease; its healing and recovery process is long. Finally, children with psychological trauma are more likely to close themselves off and may not be good at interpersonal communication when they grow up. As a result, they drift away from a good job and environment and are prone to falling into economic dilemma. Scott et al. found that violent fathers will show greater anger in the process of raising children (30), profoundly affecting children's minds and growth, including harming their health, reducing their academic performance and endangering their existence as adults (31–34). By contrast, according to attachment theory, a close relationship between the child and at least one caregiver is crucial for a positive emotional and social development (35). In the traditional “male-dominated outside, female-dominated inside” family, mothers spend more time with the children at home and develop a closer relationship with the children. Therefore, maternal emotional neglect and negative influence on children is not as serious as paternal harm to children.

We suggest it may be that adversity in early life sometimes encourages children to be independent and earned so people whose female guardians get upset or whose parents quarrel were less likely to fall into poverty as older adults. Although this finding is inconsistent with that of Repetti et al., who demonstrated that witnessing parents arguing for a long time is harmful to children' s long-term development (36), there is still an old saying in China: Loving mothers often lose children, which means the over-tolerance coddling may cause children to become ineffective, while a certain degree of adversity will be beneficial for individual development (37). Thus, the two ACEs including female guardians getting upset and parents often quarrel may encourage children to have to become independent early and develop perseverance and inner strength. It is possible for such children to overcome difficulties at work and improve their own economic conditions as they grow up.

This study has several limitations. Firstly, ACEs and depression are based on retrospective investigations and self-report scales and may be influenced by recall bias; this recall bias is also present in other studies that use CHARLS data (38, 39). However, any retrospective research has recall bias; thus, our study cannot avoid such bias. Secondly, respondents with depression may be more likely to report having experienced adversity during their childhood, whereas studies show that the measures of psychiatric treatment do not influence the report of ACEs (40). We have no effective way to verify the authenticity of the information reported by respondents. Thirdly, studies have proven a temporal association between depression and income and that change in financial hardship is associated with change in depression with time (41, 42). However, we could not testify the temporal order between depression and income measures in our study. All our data are from the CHARLS 2014, which is the only available survey that retrospectively investigates early experiences, and the CHARLS 2015. However, neither depression in 2014 nor in 2015 can be combined with income in 2015 to conduct a proper time-related analysis because they are almost in the same period. Fourth, our study can only explore whether old age depression has a mediating effect on the process of ACEs affecting poverty, rather than judging whether the association between ACEs and older adult poverty is caused by depression or income in the young age, because the CHARLS didn't retrospectively investigate depression and economic condition during respondents' youth. Despite these limitations, the results of this study could help improve our understanding of the association among ACEs, older adult poverty and depression.

## Conclusion

Paternal ACE factors make children more likely to fall into poverty as older adults, and depression has a partial mediating role on these ACE factors. We speculate that the possible reasons for these phenomena may be the father's economic status and the psychological trauma suffered by the children. Thus, evidence from this study can be used to inform policies promoting early intervention that target children at risk for adult poverty. One is to intervene in the economy. The relevant government departments should assist poor families and increase economic development. Another policy is to intervene in children's psychology, especially for people who have experienced paternal ACE events. Medical institutions and social organisations can make full use of medical resources to provide such children with psychological counselling and the treatment of depression and formulate family visit plans. As for the orphan children, it should encourage to nurture them under state supervision to protect them from life long adversaries such as depression and older adult poverty.

## Data Availability Statement

Publicly available datasets were analysed in this study. This data can be found here: http://charls.pku.edu.cn/users/sign_in/zh-cn.html. And the names of the repository/repositories and accession number(s) can be found at the above link.

## Ethics Statement

Written informed consent was obtained from the individual(s) for the publication of any potentially identifiable images or data included in this article.

## Author Contributions

HL contributed to the conception and design of the study. HL, CY, and YM conducted the data reduction and analyses. HL wrote the manuscript. JW guided the whole process and reviewed the manuscript. All authors read and approved the manuscript before submission.

## Funding

The research was supported by Research on dynamic optimization of coping strategies on health poverty risk for rural older households, funded by National Natural Science Foundation of China (Grant Number 72074086), Research on multi-dimension risk identification of health poverty vulnerability of the elderly in rural areas and targeted poverty alleviation strategy, funded by National Natural Science Foundation of China (Grant Number 71673093), Research on multi-dimension risk identification of health poverty of the elderly in rural areas and governance strategy, funded by Humanities and Social Sciences of Ministry of Education Planning Fund (Grant Number 16YJA840013). The funding bodies played no role in the design of the study and collection, analysis, and interpretation of data and in writing the manuscript.

## Conflict of Interest

The authors declare that the research was conducted in the absence of any commercial or financial relationships that could be construed as a potential conflict of interest.

## Publisher's Note

All claims expressed in this article are solely those of the authors and do not necessarily represent those of their affiliated organizations, or those of the publisher, the editors and the reviewers. Any product that may be evaluated in this article, or claim that may be made by its manufacturer, is not guaranteed or endorsed by the publisher.
